# Editorial for the Special Issue “Plant Invasions Across Scales”

**DOI:** 10.3390/plants15142170

**Published:** 2026-07-15

**Authors:** Sven Jelaska, Nina Šajna

**Affiliations:** 1 Department of Biology, Faculty of Science, University of Zagreb, Horvatovac 102A, HR-10000 Zagreb, Croatia; sven.jelaska@biol.pmf.hr; 2 Department of Biology, Faculty of Natural Sciences and Mathematics, University of Maribor, Koroška c. 160, SI-2000 Maribor, Slovenia

Plant invasion ecology examines the introduction of species, their capacity to establish, naturalize, and spread in a new region, and their interactions with native and even alien organisms in the invaded communities [1]. As primary producers and constituents of most habitats, the impacts of invasive plants can be very complex and far-reaching across trophic levels and guilds.

According to the first comprehensive global report on invasive alien species and their control, published in September 2023 by the Intergovernmental Platform on Biodiversity and Ecosystem Services (IPBES), there are 37,000 established alien species introduced by human activities around the world, with 200 new species every year [2]. Out of 3500 invasive alien species that have negative impacts on nature and humans, as many as 1061 are plants. Predictions show that new introductions will likely increase in the future [3,4]. We can look at introductions of alien plants as a continuous process with no end in sight for the near future.

Consequently, it is essential to understand how established and newly introduced alien plants cope with various environmental filters after their diaspores (seeds or vegetative parts) arrive in a new region. Additionally, we need to explore how alien plants adapt to environmental constraints in new areas and whether they will continue to spread and invade. With the ongoing influx of alien plants, the processes, mechanisms, and species interactions are becoming increasingly complex to decipher [5]. Adding to the complexity, the processes that facilitate the establishment and successful invasion of alien plants vary across scales [6], as do the mechanisms governing these processes, such as global versus local dispersal [7]. Therefore, we must identify significant processes, their mechanisms, environmental factors, and patterns across different spatial scales that deal with different phases of invasion from the individual/population/community/ecosystem perspective currently and in the future.

The Special Issue, Plant Invasions across Scales, brings together eleven contributions: ten research articles and one review that examine the mechanisms, impacts, and management of plant invasions across multiple scales and perspectives. Covering ecosystems in Europe, Asia, and the Americas, these studies employ various approaches: laboratory, common garden, and field experiments, long-term field monitoring, and modeling to provide insights into invasion processes.

From the individual and population perspective, several studies focus on morphological and physiological adaptations, biochemical interactions, and competition by studying the traits that enable invasive species to thrive in novel environments and communities. Mohammed and Mummenhoff [8] demonstrate that seed traits in invasive *Lepidium* L. species differ in drought-tolerant germination. This is further reflected in their distribution area, indicating that *Lepidium* with indehiscent fruits could persist in arid conditions. Clements and Kato-Noguchi [9] review the five major mechanisms of invasiveness of a globally notorious invader, perennial creeper *Mikania micrantha* Kunth (Asteraceae), stressing particularly its defensive capabilities among those also referring to allelopathy. Similarly, the allelopathic potential of *Heracleum mantegazzianum* Sommier & Levier (Apiaceae), studied by Gruľová et al. [10], helps the species to influence co-occurring species negatively and establish its dominance. Kreća et al. [11] investigate morphological and ecophysiological traits of how *Duchesnea indica* (Andrews) Teschem. (Rosaceae) adapts to varying light, nutrient, and competitive pressures in Europe, showing its ability to outcompete co-occurring native *Glechoma hederacea* L. (Lamiaceae) under favorable conditions. Whether competition results in species coexistence or exclusion depends on species’ competitive ability, their fitness, and community stability [12]. Xu and DeAngelis [13] model competition between the invasive macrophyte *Pontederia crassipes* Mart. (Pontederiaceae) and native submersed vegetation in shallow lakes, showing how temperature and nutrient levels mediate coexistence or exclusion under climate stress. Therefore, the coexistence of native plants with alien plants requires substantial plasticity of native plants under invasion pressure. Nikolić et al. [14] document morphological and anatomical adaptations of the European native aquatic plant *Potamogeton gramineus* L. (Potamogetonaceae) in response to the invasive *Elodea nuttallii* (Planch.) H. St. John (Hydrocharitaceae) in Vlasina Lake (Serbia, Europe).

The perspective of community dynamics and processes at the landscape level is covered by observational studies as well as predictive modeling that incorporates current distribution data into future climate models. A four-year field experimental study by Suraweera et al. [15] to restore native diversity in Bundala National Park (Sri Lanka) shows that some alien invasive plants decline naturally, while some, such as the invasive tree *Prosopis juliflora* (Sw.) DC. (Fabaceae), need to be controlled by uprooting and replanting native species. Kermavnar and Kutnar [16] use 30-year vegetation survey data of oak forests in Slovenia (Europe) to link habitat degradation, particularly oak mortality, to increased alien species invasions, with species like *Impatiens parviflora* DC. (Balsaminaceae) thriving in the understory of disturbed habitats. Climate change emerges as a key driver of range expansion [17]. De Groot et al. [18] develop risk maps for globally invasive tree *Ailanthus altissima* (Mill.) Swingle (Simaroubaceae) and tall herb *Phytolacca americana* L. (Phytolaccaceae) in Slovenia, highlighting the role of disturbances in forests and forests’ proximity to infrastructure in predicting invasion hotspots. Lal et al. [19] combine field surveys with niche modeling to project the distribution expansion at lower and higher elevations of the annual herbaceous weed *Calyptocarpus vialis* Less. (Asteraceae) in the Indian Himalayas by 2070 under future climate scenarios. Dorji et al. [20] compare mechanistic and correlative models to predict global and local suitability for establishment of *Parthenium hysterophorus* L. (Asteraceae), one of the worst weeds of ruderal and agricultural land worldwide, forecasting a 23% current suitability in Bhutan with future contractions and northward shifts under climate change, posing increased risks to agriculture.

This Special Issue highlights that the mechanisms of plant invasions operate at different scales: ecophysiological and allelochemical traits enable establishment while competition and habitat disturbances drive local dominance. Additionally, it offers insights into how predicted future climatic changes will enhance the spread and widen the distribution of invasive plants. These findings are important for developing sound predictive models, particularly those enhancing early detection and successful targeted management strategies.

We are thankful to the authors, reviewers, and editorial team for their contributions to this Special Issue, which we hope is going to inspire further research addressing processes and mechanisms of plant invasions.

## Data Availability

No new data were created or analyzed in this study. Data sharing is not applicable to this article.
